# Problems, policy and politics – perspectives of public health leaders on food insecurity and human rights in Australia

**DOI:** 10.1186/s12889-021-11188-8

**Published:** 2021-06-12

**Authors:** Stephanie L. Godrich, Liza Barbour, Rebecca Lindberg

**Affiliations:** 1 School of Medical and Health Sciences, Edith Cowan University, South West Campus, 585 Robertson Drive, Bunbury, Western Australia 6230 Australia; 2 Department of Nutrition, Dietetics and Food, Monash University, Notting Hill, Victoria 3168 Australia; 3 The Institute of Physical Activity and Nutrition (IPAN) and the School of Exercise and Nutrition Sciences, Deakin University, Geelong, Victoria 3217 Australia

**Keywords:** Food insecurity, Human rights, Public policy, Public health, Advocacy

## Abstract

**Background:**

To achieve zero hunger targets set within the United Nations’ Agenda 2030, high-income countries such as Australia must reconsider current efforts to improve food security. This study aimed to; explore perspectives from public health nutrition experts on the usefulness of drawing on the international human right to food, and associated mechanisms, to address food insecurity; identify potential roles of key stakeholders in Australia to implement a rights-based approach; and examine barriers and enablers to achieving the right to food in Australia.

**Methods:**

Qualitative in-depth interviews were conducted with key informants (> 10 years professional experience). Braun and Clarke’s (2006) six-phase approach to thematic analysis was employed to analyse data, using Kingdon’s multiple streams framework (1984) to examine interactive variables which affect policy-making processes.

**Results:**

Thirty interviews took place, with most participants representing academia (*n* = 16), majority had 10–14 years of experience (*n* = 12) and almost one quarter (*n* = 7) were in senior leadership roles. Participants believed that framing food insecurity as a human rights issue could be effective when communicating with some audiences, however alternative rhetoric is more popular and potentially more effective. Citizens, government, food industry, non-profit sector, research/tertiary and legal institutions were described as playing critical roles. Barriers to progress were identified as lack of awareness and acknowledgement of the problem, prioritisation of the private sector, lack of political will and domestic laws, and an inefficient/ineffective charitable food sector. Participants identified various enablers and opportunities for implementing a rights-based approach such as grass-roots advocacy efforts to raise awareness of the issue, integrating human rights into government frameworks and community projects and the political will to support action aligned with sustainable development.

**Conclusions:**

Human rights language and mechanisms have the potential to trigger genuine commitment to addressing food insecurity however should be used with caution. Australia’s public health workforce requires increased capacity to implement a human-rights approach and framing such efforts to align with sustainable development may achieve greater political action.

**Trial registration:**

Ethics approval was received from the Deakin University Human Research Ethics committee (project ID HEAG 168_2018).

**Supplementary Information:**

The online version contains supplementary material available at 10.1186/s12889-021-11188-8.

## Background

Worldwide, the number of people affected by hunger continues to increase [1], and progress on the Sustainable Development Goal 2 ‘Zero Hunger’ is limited. Despite the global agenda, ensuring access to safe, nutritious and sufficient food for all is unlikely to be achieved within the next decade, even in high income countries like Australia. Food insecurity - inadequate access to healthy, affordable and culturally appropriate food – has physical and mental health implications impacting both individuals and wider society. Australia’s reported prevalence of household food insecurity ranges from between 4% [2] and 18% [3] with the burden disproportionately worn by low socioeconomic status children, people who are unemployed or on low incomes, Aboriginal and Torres Strait Islander people, single-parent households, people with a disability and Culturally and Linguistically Diverse populations [4]. Despite growing evidence of their short-comings, Australia’s dominant response to household food insecurity remains government social welfare payments, and charity-operated emergency food relief. It is broadly acknowledged that these responses fail to address key determinants of food insecurity such as insecure employment and housing, and fail to ensure access to safe, nutritious and sufficient food for all [5].

As stated in the 1948 Universal Declaration of Human Rights, “*everyone has the right to a standard of living adequate for the health and wellbeing of himself and his family, including food…”.* Although not legally binding, this Declaration prompted the International Covenant on Economic, Social and Cultural Rights which was ratified by many countries, including Australia in 1975. Despite this, the right to food is not enshrined in Australian law. Particularly over the last decade, a rights-based approach to food insecurity has been proposed as an alternative to the status-quo and as a means to address the systemic, root causes of food insecurity in the high-income country context (UK, USA) [6–8] with legislative action a critical component. A recent analysis based on the Food and Agriculture Organization’s guidelines on the right to food has described what this approach could look like in the Australian context, defining six vital domains of action; national leadership, accountability and monitoring, empowerment, resourcing, non-government actors and sustainable food production and consumption [9]. A rights-based approach *“brings about a ‘root cause’ approach, focusing primarily on matters of state policy and discrimination. The move from needs to rights, and from charity to duties, also implies an increased focus on accountability”* [10].

In 2015, Australian civil society founded a ‘Right to Food Coalition’ and inspired by the first visit to an OECD country by the Special Rapporteur on the Right to Food in Canada in 2012, the Coalition attempted to arrange an in-country visit in 2019. So, whilst there is some potential momentum and good evidence for using a human rights approach to advance household food security, missing from the literature is exploration of how a rights-based approach is perceived and could be achieved, according to Australia’s experienced public health nutrition workforce. This exploration would provide a greater understanding regarding the usefulness of human rights-based framing of food insecurity, and outline a pathway towards achievement of adequate access to healthy, affordable and culturally appropriate food for all Australians, particularly from a policy perspective.

An approach that has been used to explore policy within multiple contexts, and could inform a pathway to a more food secure Australia, is Kingdon’s multiple streams framework (1984). This framework allows for the examination of interacting variables which affect the agenda setting process, namely within the following three categories of variables; (i) the *problem stream* – perceptions of the problem (ii) the *policy stream* – possible policy action and inaction, and (iii) the *political stream* – factors that may influence the body politic [11]. Kingdon’s (1984) framework suggests that these three streams run concurrently until critical points in time, when the *policy window* opens and the streams cross allowing for the policy process to attempt to address the problem. The final variable in the framework is the *policy entrepreneurs*, which are events or stakeholders that may externally trigger the opening of a policy window [11]. Collectively, the multiple streams approach enables a comprehensive analysis of the many variables at play in progressing effective public policy. Given that an understanding of the potential usefulness of a human-rights based approach to food security in Australia remains unknown, the multiple streams framework underpinned the current study which aimed to: i) explore Australian public health nutrition experts’ perspectives on the usefulness of drawing on the international human right to food, and associated mechanisms, to address food insecurity; ii) identify the potential roles of various sectors; iii) examine the key barriers of and enablers for achieving a food secure Australia; iv) uncover national and international best practice examples; and v) identify critical stakeholders or ‘policy entrepreneurs’ who could progress action towards a food secure Australia.

## Methods

### Study design and setting

A qualitative study was conducted according to a constructivist epistemology, whereby knowledge to answer our research question was created by both the researchers and participants [12]. An iterative, or hermeneutic, approach to our enquiry was adopted to continually seek meaning, particularly throughout the data analysis processes [12].

### Sample and recruitment

Eligible study participants were key informants with at least 10 years professional experience in public health nutrition and/or food security in Australia. Key informants were identified using three approaches; i) ‘Food insecurity’ scholars were identified by searching within Scopus database and Australian conference booklets for research papers with titles, key terms or abstracts including terms such as ‘food insecurity’, ‘food bank’, ‘household food security’, ‘right to food’, ‘hunger’. Example conferences included the Public Health Association of Australia’s Food Futures Conferences, Dietitians Australia Conference, Nutrition Society of Australia Annual Scientific Meeting and the Food Governance Conference. The timeframe included the past 5 years for research papers and the past 1 year for conference presentations (2019–2020). ii) Leaders and senior staff members from relevant organisations were identified via desktop analysis. Website searching involved identifying relevant government, not for profit, social enterprise, academic organisations then reviewing their mission, vision, principles, ‘what we do’ statements to ensure they sought to address ‘food insecurity’, ‘hunger’, and/or promote ‘household food security’ or the ‘right to food’. The names of individuals in relevant positions at such organisations were obtained; iii) Peer-nomination snowball sampling to identify additional eligible participants [13]. Once identified, eligible participants’ details were added to a database including name, publicly available email address, organisation name and geographic location. Seventy-seven eligible key informants (*n* = 36 academics, *n* = 26 non-government and *n* = 15 government employees) were identified.

The project team invited all participants by email and included the project description, and information letter and consent form. After the initial email, where there was no response or where a signed consent form was received by the project team, the participant was contacted via telephone to discuss the project and schedule an interview time. However, after three attempts, where there was no positive response, recruitment of that participant ceased. Of the with *n* = 77 people invited, *n* = 32 people consented to participate. Interviews took place with *n* = 30 participants including *n* = 16 academics, *n* = 8 non-government and *n* = 6 government staff.

### Interview guide development

An interview guide was developed to explore the interviewee’s perspective of whether the human right to food was a used and useful term, the interviewee’s vision of what a food secure Australia could look like, barriers to and enablers of change to achieve this vision (Supplementary Material: Interview Guide, summary in Table 1). An example question included: “*What current opportunities (such as frameworks, strategies or activities) are in place now to support achieving a human right to food in Australia in the future?”* The interview guide was pilot tested with two consenting individuals prior to use, with only minor wording changes made as a result of pilot testing. A shared understanding of the concept of food security was assumed, given that all participants had been working in the public health field for at least a decade.

**Table 1:** Summarised Interview Guide

• Can you tell me about your current work? • Health care, free speech or living a life free from discrimination are the rights of every Australian, regardless of their income, location or any other factor. Do you think that food is somehow different to these otherwise “universal” requirements? • What comes to mind when I say the term “the human right to food”? • In Australia, have you heard this term used widely? • Do you use it? If so, when and in what circumstances? • In your opining, is the ‘human right to food’ a helpful concept, even when it is not enforceable like civil and political rights are? • Imagining that every Australian is able to eat well - we have achieved a “human right to food” – just like free speech for example. • What is the government doing in this best-case scenario? • What are not-for-profit organisations doing? • What role do other major players have? For example, the food industry, research and tertiary sector, legal institutions, citizens. • Are there other important players, for example international actors? • Thinking of barriers now. What do you think are the road blocks to achieving this vision of a human right to food in Australia? • Now thinking of the enablers or opportunities for change. What current opportunities (such as frameworks, strategies or activities) are in place now to support achieving a human right to food in Australia in the future? • What’s working? What do we need to keep? Have you seen best practice internationally?	

### Data collection

The research team included one male and three female researchers in the areas of food security and sustainable food systems, who were trained in qualitative interviewing and had qualifications ranging from Honours to Doctor of Philosophy (PhD) and were in academic positions within Australian universities. Consenting individuals were believed to be motivated to participate in the project due to their interest in food security and/or human rights. Interviewers had a professional relationship with some interviewees included in this study, given their work in similar fields. Where a professional relationship existed, for example if the researcher and participant had been employed by the same organisation or co-authored research together, another researcher conducted the interview as per best-practice recommendations in qualitative data collection methods [14].

Most interviews were conducted by a trained research assistant (*n* = 25) and each author conducted at least one interview. Interviews followed a semi-structured interview guide as outlined above.

Interviews were conducted via videoconference or telephone (*n* = 29), or face to face (*n* = 1) in a location convenient to the participant with only the interviewer and interviewee/s present. Interviews were audio-recorded, with participant permission. Interviews ranged in length from 20 to 68 min and were on average 49 min long.

### Data analysis

Demographic data about interviewees was tabulated according to provide context to the perspectives presented in this study. Data was categorised and charted directly from the interview transcripts to describe the interviewees’ years of relevant experience and relevant detail about their current employment such as geographic location, sector and role. To categorise the latter, the level of leadership or influence involved in current employment was considered based on the job title provided by interviewees. Senior leadership roles included Chief Executive Officers, Board members and managers, practitioners included project officers, co-ordinators and nutritionists, senior academic included Professors, Associate Professors and Directors, early-mid career academics included senior lecturers, lecturers research fellows and PhD candidates.

Braun and Clarke’s (2006) six-phase approach to thematic analysis was employed to analyse data. Phase one included transcribing interviews verbatim. Most audio-recordings were professionally transcribed (*n* = 21), and trained research assistants transcribed remaining interviews (*n* = 9). Transcripts were double-checked for consistency. Audio-recordings and transcripts were reviewed and “face sheets” for all transcripts were created to familiarise the researchers with the data [12]. Summary data included on the face sheets included interviewee name, length of interview, special circumstances or contextual issues which might impact the data, major issues emerging, and issues requiring follow up. A cloud-based shared drive was used to maintain an ‘audit trail’ of all documents and steps taken throughout the study. This strategy ensured dependability and confirmability [15].

All transcripts were imported into QSR NVivo 12 Plus for analysis; and All authors and a trained RA read and re-read the interview transcripts and face sheets to immerse themselves in the data. Initial codes were developed and added to a coding frame document in Phase two, using a semantic, realist approach. The coding frame included each node’s title, description of content coded and an exemplar quote. Phase three included combining nodes to form categories and then overarching themes, the latter derived from Kingdon’s Multiple Streams Framework [11]. Kingdon’s (1984) framework allows exploration of interacting variables which affect agenda setting. As this research is intended to describe enablers and barriers to achieving progress on the right to food whereby public policy is a key mechanism, this framework was used to guide data analysis. Deductive analysis involves coding and theming the data against pre-determined concepts [16], in this case, the four theme domains presented in Kindon’s Multiple Streams Framework [17]. A project map was generated via NVivo to visualise how codes and categories were embedded within themes. During phase four, one author reviewed all coded extracts within each theme. To increase credibility, a second team member cross-checked coded themes [15]. The whole dataset was then reviewed to verify each theme’s inclusion in the dataset and thematic map. Phase five included generating a definition of each theme to convey its ‘essence’. The final phase, six, included writing a concise story to represent the data. To ensure transferability, a thick description was used to describe the context of interviewee experiences [15]. Saturation was confirmed when no new themes were created in the NVivo database. The consolidated criteria for reporting qualitative research (COREQ) checklist [18] was utilised to design the study and report on the methods.

Ethics approval was received from the Deakin University Human Research Ethics committee (project ID HEAG 168_2018).

## Results

Of the thirty interviewees, most were employed by academic institutions (*n* = 16, 53%), had 10–14 years of experience (*n* = 12, 40%) and were located in Victoria (*n* = 12, 40%) as outlined in Table 2. One in five interviewees worked in government departments or organisations and almost one quarter were in senior leadership roles.

**Table 2:** Demographics of interviewees

	*Frequency (n = 30)*	*Percentage (%)*
Current employment		
Academic	16	53
Non-Government	8	27
State Government	5	17
Local Government	1	3
Current role		
Senior Leadership	7	23
Practitioner	9	30
Senior Academia	5	17
Early-mid Career Academia	9	30
Years of relevant experience		
10–14	12	40
15–19	7	23
20–29	5	17
30+	5	17
Unassigned	1	3
State/Territory of Employment		
Victoria	12	40
New South Wales	5	17
Queensland	5	17
Western Australia	4	13
Tasmania	3	10
South Australia	1	3
Northern Territory	0	0
Australian Capital Territory	0	0

To answer the research questions, data were organised into five overarching themes, derived from Kingdon’s Multiple Streams Framework (1984):

i. The problem stream – how is the ‘problem’ of food insecurity, and the ‘right to food’ concept received and defined in Australia?
ii. The policy stream – what role do key stakeholders have in proposing feasible solutions to achieving a human right to food in Australia?
iii. The political stream – how does the political context present enablers and barriers to respect, protect and fulfil the right to food in Australia?
iv. Policy windows – what do opportunities for policy action look like in Australia, and what are some best practice international examples we could draw upon?
v. Political entrepreneurs – who are the key players to identify and action opportunities?

### The problem stream

In exploring the problem of food insecurity, participants were prompted to consider whether or not human rights language was useful to achieve progress and whether it is commonly used in practice.

#### Framing food insecurity within human rights terminology is effective when communicating with some audiences

Participants described that the usefulness of the term *human right to food* depended on the intended audience. Participants agreed that different language appeals to different audiences and suggest that human rights language may hold more power when engaging political decision-makers, particularly in states and territories of Australia which have human rights charters or legislation. However, some participants suggested this terminology be used with caution, advising that under Australia’s current leadership there is not much of an appetite for human rights discourse. Human rights language was described as being associated with issues such as people seeking asylum in Australia and Indigenous affairs, where the framing has had limited perceived success. The term *human right to food* was described as prohibitive, polarising, and academic by some participants.

*“I think maybe human rights is used by some as, I don't know, a lefty agenda and it has become a bit polarised in that way.” (Academic, Senior Academic, 30 years)*

The term *human right to food* was described as being somewhat utopian, in terms of making food security a legislated issue, and therefore of limited usefulness to achieve real-world progress. Participants suggested alternative terminology to engage specific audiences. Some participants advised that based on evidence about values-based communication, terms such as ‘deserves’ and ‘fairness’ are more effective than ‘rights’ in engaging everyday people. Participants who described these terms, did so within the context of reflecting Australian values whereby every living human in Australia ‘deserves’ access to adequate food to maintain a healthy life. In order to gain traction, civil organisations and individuals must be empowered to advocate and to do so, the issue must be framed with more publicly palatable language. The right to food framing may be helpful in engaging policymakers, academics and legal audiences, however to engage community members terms such as ‘food sovereignty’ in response to issues of ‘food stress’, ‘poverty’, ‘cost-of living’ are, according to interviewees, more effective.

#### Human rights language presents a novel framing for the public health workforce; however, progress is required to define this approach and ensure accountability

Some participants viewed the term as presenting a novel frame for public health professionals on the long-standing issue of food insecurity, offering a sense of optimism to achieve progress. The term was described to eliminate problematic ‘victim blaming’ associated with many approaches to address food insecurity and it challenges society and policymakers to consider their social conscience. Several participants also believed it could be a useful term to use when linking to other broader human rights agendas and mechanisms, such as the 2030 sustainable development agenda which include many references to human rights and the Australian government is committed to meeting and reporting on targets around zero hunger. There is also value in framing food security as a moral issue to build public pressure and shame the government to act.

However, until accountability to achieve the human right to adequate food is enshrined in domestic legislation, for example via a National Charter of Human Rights, participants believe there is limited value in using such framing to achieve progress.

*“We would need to see a Food Security Act from the Commonwealth Government and that would mean that if a person in Australia didn’t have food security they would have a potential remedy or an enforceable right, whereas now it is just an aspirational right.” (State Government, Practitioner, 20 years)*

There is also work to be done to define what the ‘right to food’ actually looks like in practice. Participants described the importance of food meeting specific cultural needs and reinforced that when individuals are required to source their food from emergency relief services, their human right to food is not being fulfilled.

#### The term ‘human right to food’ is seldom used in the public health rhetoric

Although potentially useful to engage stakeholders from sectors beyond public health, the existing food security, nutrition and welfare workforce are not consistently using the term *human right to food*. Many participants recalled that they’d only started hearing it used or using it themselves in the last couple of years. Participants recalled the phase being used most commonly when referring to remote Aboriginal communities and people seeking Asylum where the issue of food insecurity is more commonly understood by the general public.

*“This is not the language of practical or practitioner policymakers, the rights-based language – we’re not using it. We’re talking about a sustainable development goal [instead]” (State Government, Senior Leadership, 32 years)*

Participants advised that in Australia’s current neoliberal political climate, policymakers are not prioritising the protection of rights, unless it’s framed within the 2030 sustainable development and attributed to targets that have already been committed to. Participants suggest that training and support is required to ensure its utilisation is effective and appropriately targeted to specific audiences, as human rights terminology is not part of traditional training across these sectors. In building workforce capacity, training must link human rights to existing policy agendas and explain current legislative accountability.

*“I think we’ve got to start slipping it into conversation rather than marching forward with banners, if you know what I mean” (Academic, Early-Mid Career Academia, 17 years)*

Most participants agreed that the human rights discourse adds importance to the issue of food insecurity, and that such language and the associated frameworks, could amplify public health advocacy efforts.

### The policy stream

Participants described actors’ potential roles and responsibilities if achieving a human right to food was ensured for everyone in Australia, as outlined below by actor type:

#### Government

Key government roles included reducing income inequality through strategies like a Universal Basic Income, increases to social security benefits and state pensions and increasing the minimum wage. Respondents envisaged alternative, affordable food systems where there was no supermarket ‘duopoly’, and food was free from commercial interference. A Department of Food would support interdepartmental plans and policies including a National Food Plan and poverty reduction strategies, i.e.:

*“We need some type of blueprint that the governments at each level have committed to…. Centrelink payments … Newstart payments, so that needs to be increased.” (Academic, Early-Mid Career Academia, 10-14 years).*

#### Food industry

Minimising the market-driven approach to food industry was reinforced by some participants, as was reducing junk food advertising, particularly among children. One participant envisaged a reduction in ‘big food’ sponsoring sporting clubs, others a focus on Corporate Social Responsibility, i.e.:

*"We would need legislative provisions that stopped food corporations from selling unhealthy products and behaving in a way that compromises full food security in the pursuit of profits for shareholders." (Academic, Early-Mid Career Academia, 10–14 years)*

Some participants envisaged supermarkets amending their logistics systems to ensure there was no surplus food being redistributed to the charitable food sector. The role of supermarkets in ensuring fair food prices for Australian farmers was discussed by two participants, for example, ensuring farmer contracts supported their livelihoods. Importantly, in remote communities, food outlets would be run as a community service rather than maximising profitability.

#### Not-for-profit sector

Some respondents proposed the charitable food sector develop surplus food donation guidelines, to reduce the current *“dumping ground”* and *“band aid”* approach for supermarkets or companies and improve the nutrient density of food available to vulnerable community members. Some believed the charitable food sector had no place at all. Respondents described non-government organisations as *“connectors”, “navigators”, “amplifiers” and “advocates”* between government and community, and giving a voice to community members often unheard, i.e.:

*“Providing them with access to on the ground experiences that people are facing. Often they’re quite good at translating these real-life stories to policy makers … they have a great role there in ensuring that any policy that’s being decided upon …is going to be as impactful as possible.” (Academic, Senior Academia, 15-19 years).*

Other respondents described a truly unified and collaborative sector where all programs had a human rights lens, while others still outlined a successful social entrepreneurship approach that accounted for various contexts.

#### Research and tertiary sector

Embedding human rights education into tertiary curricula was outlined by academic participants. Some believed there was an adequate evidence base regarding the problem of food insecurity, whereas limited research focused on solutions. Research across the food supply chain was perceived as important, to adequately capture the various actors’ perceptions. The research sector was envisaged to work closely with government in a food insecurity monitoring and evaluation capacity. ‘Research leadership pathways’ were described by one participant; people with lived experience were envisaged to co-produce food insecurity problem and solutions-focused research. The logistics of research projects to equip practitioners with timely knowledge was also reinforced, i.e.:

*“Universities are too slow… sharing that data that’s been gathered in a really rigorous way in a more timely manner for practitioners and policymakers.” (Non-government, Senior Leadership, 20–29 years)*

#### Legal institutions

The legal sector was seen to educate, advocate for and enforce a human rights approach to food. Legal professionals would assist with the framing of human rights terminology as well as the practicality of integrating and overseeing the approach within constitutional rights, i.e.:

*“I’m sure that legal people could be very helpful for us in terms of how you frame it and then advocacy … I think that if you want to change the paradigm, you’re gonna need advocates and they’re gonna need to be at all levels.” (State Government, Practitioner, 30+ years)*

#### Citizens

Grass roots bottom up action, driven by citizens, was commonly mentioned by participants. This included supporting community-based food programs, conveying their lived experience and *“driving a political agenda.”* Citizens also played a critical role in holding government to account, i.e.:

*“We need citizen groups to make sure that, that you know the good work is kept up. And legally, there should be, there should be legal consequences for people failing to live up to their, companies living up to their social contract.” (State Government, Senior Leadership, 15–19 years)*

#### Other actors

Public health practitioners and unions were perceived as key stakeholder groups in advancing a human rights-based approach to food. The media would play a critical role in conveying food security information, schools would ensure children had access to nutritious food, while philanthropic funders needed to be considered, i.e.:

*“The philanthropic funders and the other – the funders who did a bit outside the square of government as well and how we bring them in ‘cause again, while we have well-intentioned providers, we also have well-intentioned funders.” (Non-government,* S*enior Leadership, 20–29 years)*

### The political stream

Participants outlined positive and negative factors that could influence achievement of a right to food in Australia, presented below in the form of barriers and enablers.

#### Barriers to achieving a human right to food in Australia

There is a lack of awareness and acknowledgement of the problem. A human rights approach to food was not of concern to many people, including politicians, with issues like obesity *“getting the limelight”*. A lack awareness of the human right to food at a practitioner, management and organisational level was reported, with a human rights approach portrayed as misaligned with strategic plans and policies. Some respondents attributed this with differing worldviews, one referred to a lack of common language describing the problem, while others believed it was a mindset of a *“business as usual”* or *“out of sight, out of mind”* approach, particularly in large geographical regions. Some respondents firmly believed the lack of government leadership, at multiple levels, was to blame for a lack of advancement of household food security, with an individual responsibility narrative common and a perception that the problem was *“too hard to fix”*. Many participants described the lack of nationally representative household food security data as a barrier to shining a light on the issue, i.e.:

*“We’re not monitoring it using an appropriate measure, we’re not monitoring it consistently, we’re underestimating it, we’re saying it’s not such a big problem, so let’s brush it under the rug. So, that is one of the biggest barriers, the fact that we just, we don’t want to know” (Academic, Early-Mid Career Academia, 10-14 years).*

Private sector profits are being prioritised. Given the government’s focus on individual responsibility, the ‘onslaught’ of cheap, processed food by an industrialised food industry reportedly led to *“default purchases”* of unhealthy food with limited regulation. Numerous respondents described the issue of farmers paid too little, or contractually obliged to only sell to the major supermarkets, and in the situation where such supermarkets wouldn’t take the produce, it was wasted, i.e.:

*“The absolute power they have over our food systems … the distribution chain and the farmers and how much they are controlled by what major supermarkets demand of them and I think if they changed their value set then we would be in a really, really different place.” (State Government, Practitioner, 10–14 years)*

Some respondents believed we have sufficient information to advance a right to food, but political will and domestic laws to protect this right, are lacking. The neoliberal, individualistic ideology of government was reinforced by many respondents, with food *“seen as a consumer product rather than a public service”* in the current deregulated environment*.* Other commentary focused on Australia’s reductionist society. One respondent described local government as having *“their hands tied by state policy”,* citing challenges to restrict fast food. Policy changes to several government programs including the community development program, resulted in remote community members *“missing out on income”* due to changing regulations, impacting their ability to purchase food. The lack of enforceable human rights law was consistently described by participants as highly problematic, i.e.:

*“No one has got a right to food security…if I was on welfare and they put me on the cashless welfare card up in the Northern Territory, I can’t take the minister for social security to court and say this breeches my right to food security because they would go oh yes, and what right is that, what right is being breeched, you show me the Act of parliament… I’d just have to point to international law and that’s not an enforceable right” (Academic, Early-Mid Career Academia, 10+ years)*

The existing charitable food sector was described as inefficient and ineffective. Service duplication and a siloed working approach in a *“corporatised”* charitable food sector was criticised by some interviewees, as was the pushing of organisations’ agendas on service recipients. The unsustainable model of distributing donated food was discussed; resources were described as *“stretched”* with organisations lacking time to explore other solutions to the current model that would improve livelihoods long term, i.e.:

*“We're at risk of just creating this big machine that feeds insecure people and doesn't have any mechanisms in which to actually reduce how many people are food insecure.” (Non-government, Practitioner, 15–19 years)*

#### Enablers and opportunities for achieving a human right to food in Australia

Advocates are raising awareness about this issue. Social justice organisations with articulate media spokespeople were promising to *“appeal to hearts and minds”,* while the suggestion to *“nudge things rather than revolutionise things”* was made by one respondent. Remote community leaders were seen to be important advocates for their own communities to seek out desired information and action. Several interviewees referred to a collective approach to advocacy, such as through civil society organisations, as an opportunity, i.e.:

*“There’s real opportunities to develop really clear strong messages for government and also to really work on what are the key research questions… I feel like there’s a potential to really harness a group, that a contingency, that are working more and more together” (Academic, Early-Mid Career Academia, 20-29 years).*

Integrating human rights further into government frameworks and community projects is possible. Some interviewees suggested incorporating human rights language into existing strategies, such as the National Obesity Strategy or proposed new National Preventive Health Strategy, given the alignment between issues. Successful community-based approaches were described within a broader approach as empowering community members and required longer term investment, i.e.:

*“I think community food co-operatives are probably something that is going to get more attention because of the combined effect of [the] bushfires and Coronavirus, so perhaps there’s scope for expanding local or state government support for, for local food co-ops.” (Academic, Early-Mid Career Academia, 10–14 years)*

Capitalising on the acceptance and action on the Sustainable Development Goals was also perceived as important, with human rights approaches rather than the terminology being adopted into such action. A more recent focus on sustainable economy strategies was perceived as having potential to create dignified opportunities to feed vulnerable Australians. *“Localised food systems”* were increasing in popularity, as were food alliances and Food Policy Councils which had seen cross-sector collaboration to address food system issues. These governance strategies were *“mechanisms for collaboration”,* i.e.*:*

*“If we can integrate all of these projects and these bodies and we can take a holistic and systemic approach, then we're in a really good place to really begin to mainstream food poverty as an issue and to start to look at what we need to do to really alleviate it.” (Non-government, Practitioner, 14-19 years).*

### Policy windows

In addressing the research aim to uncover national and international best practice examples, participants were asked to describe best practice examples from Australia and overseas, where efforts towards achieving the human right to food have been successful. Participants described a range of strategies which they believed to have been effective, as categorised in Table 3 into social welfare policy, legal frameworks, community-led approaches and food distribution platforms. These strategies present potential ‘policy windows’ or action items which could be used by advocates to advance a right to food in Australia. Detailed exploration of the efficacy and feasibility of the implementation of these strategy options beyond their current region was beyond the scope of this study.

**Table 3:** Australian and international exemplars of strategies to achieve the human right to food

Strategy	Australian example	International example
Social welfare advocacy or policy	• Raise the Rate (Newstart) • Right to Food Coalition (National)	• Norway • Sweden • School Lunch Program (Japan) • Basic Pension Policy (New Zealand) • Canada – Paddock to Plate (British Columbia) • National Food Policy (Brazil) • Women, Infants, and Children (WIC) program (USA) • Right to Food Scotland Bill (Scotland)
Legal frameworks	• Charter of Human Rights (Victoria)	
Community-led approaches	• Remote food symposium (Northern Territory)	• Food Lab Detroit (USA) • Vibrant Communities (Canada) • Incredible Edible (UK)
Food distribution platforms and models	• Open Food Network (National) • Asylum Seeker Resource Centre Food Justice Truck (Victoria) • Melbourne ‘pay as you feel’ restaurants (Victoria)	• The Stop (Canada) • Food Hubs (USA) • Food growing on urban building roofs (France)

### Political entrepreneurs

As articulated above, participants referred to various key stakeholders or ‘policy entrepreneurs’ as being pivotal to progressing action towards a food secure Australia and described whether a human right framing of the issue was currently being or could potentially be used to influence policy and practice. These policy entrepreneurs could link “*policy problems and policy solutions together with political opportunities*” [19] and were described by participants as representing government, not-for-profit, food industry, legal and tertiary sectors. Based on our analyses, key potential entrepreneurs that could be utilised to progress a human rights agenda in Australia are listed below:

- Government representatives such as Members of Parliament, Local Government Councillors or employees could advocate within parliament to advance policy actions.
- Not-for-profit sector staff such as charitable organisation staff or volunteers could act as connectors between government and community, representing and advocating for community needs.
- Tertiary sector staff such as researchers could be used to publish research on how the problem of food insecurity could be best framed for maximum impact, and lecturers could include human rights-based approaches in their tertiary education courses.
- Legal professionals such as lawyers could support other sector stakeholders to frame food security using correct human rights language and aid its integration into constitutional rights.
- Citizens are urged to hold the government to account
- Public health practitioners could utilise human rights language if engaging in food security advocacy.
- The media could similarly adopt human rights language in conveying food security information.

## Discussion

This study aimed to: i) explore Australian public health nutrition experts’ perspectives on rights-based approach to addressing food insecurity; ii) identify actors roles and responsibilities in the local context; iii) examine potential barriers to and enablers of achieving the right to food; iv) uncover best practice examples; and v) identify policy entrepreneurs who could progress action. Our study findings suggest that human rights language, while novel and starting to be used more in the Australian public health context, may not be widely useful for improving food security in Australia currently. The framing of the issue using such terminology was perceived to have little sway over policymakers and was seldom used outside of academia and legal sectors. In contrast, terms including ‘deserves’ and ‘fairness’ were perceived as more effective than ‘rights’ in engaging everyday people, possibly given that these terms are more commonly used. In order to gain traction, the issue must be framed with more publicly palatable language. However, it was clear that amongst participants, a broad consensus exists about what a human rights-based approach to food security stands for and the importance of achieving this. Advocacy and action to embed attributes of human rights into frameworks, policy documents and organisational actions should hence continue and be possible in this context.

To achieve a food secure Australia, study respondents suggested the government should lead legislative changes, the not-for-profit sector should act as connectors between government and community members, the research sector could support food insecurity monitoring and evaluation, legal professionals should assist with the framing of human rights terminology and citizens should drive the political agenda, holding government to account. However, barriers to change included a lack of wide-spread awareness of what a human rights-based approach to food security is, lack of enforceable human rights law as a mechanism for change, and a siloed working approach in a *“corporatised”* charitable food sector. In contrast, enablers included strong social justice advocates, community empowerment and capitalising on the acceptance and action on the 2030 Sustainable Development Goals.

Our findings regarding the usefulness of a human rights-based framing corroborate international literature. Human-rights framing is already in place in legal and political sectors, but as Freeman (2017) argues, *“we must distinguish the human rights from the legal rights of particular societies”.* While human rights-based rhetoric provides a mechanism to *“reframe ‘problems’ as ‘violations”’* and presents a stronger argument that it should not be tolerated [20], Chilton and Rose (2009) assert that in order to be effective, a clear consensus on the definition of the ‘right to food’ is required. Further justification for the theory of human rights and why this framing should be used is required to convince stakeholders to support this language [21].

The vision of a food secure Australia painted by our respondents makes clear how actors can implement a rights-based approach and their insights appear well aligned with activities internationally. Freeman (2017) outlined that while governments create politically-motivated laws, numerous political factors influence the extent to which they implement human rights approaches. A report by the Special Rapporteur [22] to the Canadian government in 2012 recommended that a national food strategy be written and an increase of the minimum wage to ensure an adequate standard of living [21], both of which are in line with our interviewees’ calls for a National Food Plan and increased income equality in Australia. Non-government actors, across the globe, have been urged to monitor human-rights based activities and performance by governments, and citizens urged to mobilise their power [10] Participants in our study suggested that translating ‘real-life stories to policy makers’, ‘connecting’, ‘advocating’ and tailoring interventions to the local context, are some of the tangible activities required for human-rights based approach.

Barriers and enablers outlined by our interviewees also reflected the international literature. For example, respondents in our study described a lack of awareness of food insecurity as an issue in Australia by the government, particularly due to a lack of appropriate monitoring of the issue. The Canadian government has similarly been urged to appropriately measure food insecurity through the national Census [22]. Within Australia, private sector profits are prioritised, leading to substantial marketing of energy-dense, nutrient-poor food. This is similarly an issue in Canada, with food labelling and nutrition policies likewise called for [22]. Interviewees from research conducted in the UK echoed these concerns, indicating the profit-driven nature of supermarkets hampered potential collaboration to improve poverty [23]. The unsustainable model of distributing donated food in Australia through the charitable food sector was criticised by most of our participants. In the UK, increasing reliance on food relief and charitable providers has been controversial in nature with criticisms including reliance on an uncoordinated and overworked support sector rather than effective policy measures [24]. Enablers for achieving a human right to food in Australia cited in our study included a collective approach to advocacy, such as through civil society organisations. Similarly, in the UK, advocates have argued that there is considerable opportunity for non-government organisations, health professionals and academics to contribute to effective change [23]. Local food systems were described by our interviewees as making a promising contribution to food security in Australia. Similar recommendations have been made in Canada to grow local agricultural sectors [22].

Limitations of this study include a lack of representation of respondents from the Northern Territory, Australian Capital Territory and one respondent from South Australia. Almost half of our respondents were from Victoria which may have influenced the data due to specific state-based approaches being used. Further, we had limited participation by government participants, particularly at the national level. Study strengths include a focused, in depth exploration of the topic among experienced Australian public health professionals who have a pivotal role in advancing household food security and the integration of the Multiple Streams Framework to understand and interpret the data.

This study adds real-world strategies for practitioners and policy-makers to implement in the Australian context, with high relevance for comparable contexts internationallyProfessionals working across different sectors could utilise these findings to inform their work and advocacy for policy change. As such, recommendations include:

- At the current time advocates should use human rights language with caution, considering using more engaging terms such as ‘deserve’ and ‘fairness’ within the Australian context. An approach likely to be more successful is to anchor advocacy efforts within the 2030 Agenda/Sustainable Development Goals, which are already politically palatable.
- Workers across sectors should consider assuming the roles suggested by this study in their practice and policy endeavours.
- All actors should continue to apply pressure to the government to adequately and regularly measure food insecurity in Australia in order to better understand the true scale of the issue and advance advocacy efforts to address it.
- Sectors should incorporate human rights strategies and principles (even if not labelled as ‘human rights approaches’) into plans and frameworks to ensure equitable access to affordable, nutritious food.

## Conclusions

A rights-based framing of food insecurity is no panacea for complex challenges facing our current food system, however, the approach has the potential to enable a genuine commitment to reducing food insecurity rather than accepting the inevitability of this pressing social issue [25]. Whilst rhetoric of ‘human rights’ may have limited use in Australia currently, key informants have advised that this language is being used more recently. Public health leaders involved in this study demonstrated that the path to improving food insecurity in Australia is defined and that this approach aligns with the principles of human rights in terms of addressing structural issues through evidence-based government action and accountability. Public health leaders in this study mostly agreed upon which policy windows and entrepreneurs are critical to achieve this action. Trans-disciplinary sectors must work collaboratively to ensure human rights strategies are incorporated into government agendas to ensure progress is made to address food insecurity in Australia and contribute to achievement of the United Nations’ Agenda 2030.

## Supplementary Information

**Additional file 1.** Semi-structured interview guide.

## Data Availability

The datasets generated and analysed during the current study are not publicly available due to the potential to compromise individual privacy but are available from the corresponding author on reasonable request.

## Abbreviations

ICESCR: International Covenant on Economic, Social and Cultural Rights
PhD: Doctor of Philosophy
COREQ: Consolidated criteria for reporting qualitative research
WIC: Women, Infants, and Children Program
USA: United States of America
UK: United Kingdom
