# Supplementary material for: Problems, policy and politics – perspectives of public health leaders on food insecurity and human rights in Australia
Source: BMC Public Health. 2021 Jun 12;21:1132. doi: 10.1186/s12889-021-11188-8 (PMC8197601; doi:10.1186/s12889-021-11188-8)
Supplement: Supplementary file 1 — Additional file 1. Semi-structured interview guide. [file 12889_2021_11188_MOESM1_ESM.docx]

**Supplementary Material**

Title: ***Problems, Policy and Politics – Perspectives of Public Health Leaders on Food Insecurity and Human Rights in Australia***

Authors: Stephanie L Godrich^1^, Liza Barbour^2^, Rebecca Lindberg^3^

1. School of Medical and Health Sciences, Edith Cowan University, South West Campus, 585 Robertson Drive, Bunbury, Western Australia 6230, Australia.
2. Department of Nutrition, Dietetics and Food, Monash University, Notting Hill, Victoria 3168, Australia
3. The Institute of Physical Activity and Nutrition (IPAN) and the School of Exercise and Nutrition Sciences, Deakin University, Geelong, Victoria 3217, Australia

Corresponding Author: Liza Barbour [liza.barbour@monash.edu](mailto:liza.barbour@monash.edu)

Supplementary material:

- Semi-structured Interview Guide

**Semi-structured interview guide**

In short, the purpose of these interviews is to gather the perspectives on the human right to food from individuals, like yourself, who have worked in the food security or public health nutrition space for more than a decade.

1. Can you tell me a bit about your current work?

2. So to set the scene for this interview a bit…. health care, free speech or living a life free from discrimination are the rights of every Australian, regardless of their income, location or any other factor. Do you think that food is somehow different to these otherwise “universal” requirements?

*2a. Prompt:* ***Why*** *or why not?*

3. What comes to mind when I say the term “the human right to food”?

*3a. Prompt: in Australia, have you heard this term used widely?*

*3b Prompt: Do you use it? When / where?*

*3c.* *Prompt: in your opinion, is the ‘human right to food’ a helpful concept, even when it is not enforceable like civil and political rights are?*

4. Now I would like you to **imagine** that every Australian is able to eat well. Imagine a best-case scenario that we have achieved a “human right to food” – just like free speech for example.

*4a. Prompt: In your vision, what is the* ***government*** *doing in this best-case scenario?*

*4b. Prompt:* *what are* ***not for profit organisations*** *doing in this best-case scenario?* What is their role?

*4c. Prompt: what about some of the other major players like the* ***food industry,*** *the* ***research and tertiary sector****,* ***legal institutions, citizens….*** *any comments on what they are doing to support the human right to food in Australia in this best-case scenario?*

*4d. Prompt – are there any other important players? For example, International actors?*

5. I want to turn to barriers for change now. What, do you think, are the road blocks to achieving this vision of a human right to food in Australia?

6. Now I’d like to cover enablers or opportunities for change. What current opportunities (such as frameworks, strategies or activities) are in place now to support achieving a human right to food in Australia in the future?

*6a. Prompt - What’s working? What do we need to keep?*

*6b. Prompt – Have you seen best practice elsewhere in other countries, that you think we could learn from and create here in Australia in order to achieve a human right to food?*

7. Finally, the research team has reviewed some of the literature on this topic and we have devised a Figure to synthesise a potential road map for Australia, to achieve a human right to food. These interviews will help further flesh out the detail.

I sent you the figure and a literature review synthesis of our findings, so just hoping you can have that in front of you now…

Go through key synthesis doc – high level summary

I’ll just give you a few moments to have another read of it…

Is there anything you would add, take-away, amend or emphasise with this synthesis or figure?

*7a. Prompt: How similar/different does it look to what you outlined above when I asked you about what the right to food might look like?*

8. Is there anything else you’d like to add about what we’ve discussed today?

9. So to finish up, I’m just going to quickly run through some demographic questions about yourself just for our record.

*9a. Prompt: How many years have you worked in your field/industry?*

*9b. Prompt: In what state or territory do you work in?*

*9c. Prompt: What is your job title and name of your organisation?*

*9d. Prompt: Is there any person that you might recommend that would be interested in this study and you think we should also interview? (send follow up email)*

Thank you for your time.
